# Supplementary material for: An open-source framework for large-scale, flexible evaluation of biomedical text mining systems
Source: J Biomed Discov Collab. 2008 Jan 29;3:1. doi: 10.1186/1747-5333-3-1 (PMC2276192; doi:10.1186/1747-5333-3-1)
Supplement: Additional file 1 — Evaluation platform source code. The additional file contains the software evaluation framework discussed in this paper. The most current release can be downloaded from SourceForge.net [1]. See the accompanying README file for instructions on installation and use. Also included in the distribution is a collection of UIMA wrappers for some commonly used BioNLP tools and annotated corpora. [file 1747-5333-3-1-S1.zip › bionlp_uima_repository-1.0.1/docs/api/edu/uchsc/ccp/uima/util/opendmap/DMAPJcasTokenizer.html]

DMAPJcasTokenizer (Center for Computational Pharmacology Util/UIMA API)


|  |  |  |
| --- | --- | --- |
| **Overview** \| **Package** \| **Class** \| **Tree** \| **Deprecated** \| **Index** \| **Help** | |  |
| **PREV CLASS**   **NEXT CLASS** | **FRAMES**    **NO FRAMES**     **All Classes** |  |
| SUMMARY: NESTED \| FIELD \| CONSTR \| METHOD | DETAIL: FIELD \| CONSTR \| METHOD |  |


---


## edu.uchsc.ccp.uima.util.opendmap Class DMAPJcasTokenizer

```
java.lang.Object
  edu.uchsc.ccp.opendmap.DMAPTokenizer
      edu.uchsc.ccp.uima.util.opendmap.DMAPJcasTokenizer
```

**All Implemented Interfaces:**: Iterable<edu.uchsc.ccp.opendmap.DMAPToken>, Iterator<edu.uchsc.ccp.opendmap.DMAPToken>

---

``` public class DMAPJcasTokenizer extends edu.uchsc.ccp.opendmap.DMAPTokenizer ```

Helper class to get all of the appropriate annotation structures
out of the JCas and return them as DMAPToken structures
for the OpenDMAP parser.
There are two types of annotations of interest at the moment:

- CCPTokenAnnotation structures that correspond to individual words and pieces of punctuation- ClassMentionAnnotation structures that correspond to "named entities"

**Author:**
:   R. James Firby

---

**Constructor Summary**

`DMAPJcasTokenizer(edu.uchsc.ccp.opendmap.Parser parser,
org.apache.uima.jcas.JCas jcas,
AnnotationMap annotationMap,
boolean debug)`
  
          Create a new iterator over structures in the JCas.


| **Method Summary** | |
| --- | --- |
| `boolean` | `hasNext()`             Check if there are any more tokens to process |
| `edu.uchsc.ccp.opendmap.DMAPToken` | `next()`             Grab the next token and return it. |

**Methods inherited from class edu.uchsc.ccp.opendmap.DMAPTokenizer**

`iterator, remove`

**Methods inherited from class java.lang.Object**

`clone, equals, finalize, getClass, hashCode, notify, notifyAll, toString, wait, wait, wait`

**Constructor Detail**

### DMAPJcasTokenizer

```
public DMAPJcasTokenizer(edu.uchsc.ccp.opendmap.Parser parser,
                         org.apache.uima.jcas.JCas jcas,
                         AnnotationMap annotationMap,
                         boolean debug)
```

:   Create a new iterator over structures in the JCas.

    **Parameters:**: `parser` - The parser that will be using this tokenizer: `jcas` - The JCas holding the annotations to take as input: `annotationMap` - The map from JCas annotations to words and DMAP Protege Frames: `debug` - Print out additional debugging information if true


**Method Detail**

### hasNext

```
public boolean hasNext()
```

:   Check if there are any more tokens to process

    :   **Specified by:**: `hasNext` in interface `Iterator<edu.uchsc.ccp.opendmap.DMAPToken>` **Specified by:**: `hasNext` in class `edu.uchsc.ccp.opendmap.DMAPTokenizer`

---


### next

```
public edu.uchsc.ccp.opendmap.DMAPToken next()
```

:   Grab the next token and return it. The tokens are in a
    list sorted by ending character (which roughly corresponds to
    input token order).

    :   **Specified by:**: `next` in interface `Iterator<edu.uchsc.ccp.opendmap.DMAPToken>` **Overrides:**: `next` in class `edu.uchsc.ccp.opendmap.DMAPTokenizer`


---


|  |  |  |
| --- | --- | --- |
| **Overview** \| **Package** \| **Class** \| **Tree** \| **Deprecated** \| **Index** \| **Help** | |  |
| **PREV CLASS**   **NEXT CLASS** | **FRAMES**    **NO FRAMES**     **All Classes** |  |
| SUMMARY: NESTED \| FIELD \| CONSTR \| METHOD | DETAIL: FIELD \| CONSTR \| METHOD |  |


---
